# The COVID-19 pandemic: some thoughts on integrity in research and communication

**DOI:** 10.1080/20961790.2021.1980953

**Published:** 2021-11-09

**Authors:** David Morens, Zoë Hammatt

**Affiliations:** a National Institute of Allergy and Infectious Diseases, National Institutes of Health, Bethesda, Maryland, USA; b University of Hawaii and Z Consulting, LLC, Honolulu, Hawaii, USA

Principles of integrity and ethical practice in research have been reinforced and honed over decades. In medical and biomedical fields, they have been shaped by challenges that include endemic, emerging, and re-emerging diseases [1], as well as the scientific responses to each disease as it occurs. These responses include clinical and community prevention trials, vaccine and drug trials for persons with life-threatening diseases, applied and basic research with dangerous microbial agents, and many others. Unexpected fatal pandemics caused by novel agents—for which neither drugs nor vaccines exist—present additional urgent challenges for scientists, defined here to mean biomedical, medical, and public health scientists and practitioners. In addition to conducting research, scientists are also often tasked with communicating research findings, not only to those who make policy decisions, but also to the media and the public.

The COVID-19 pandemic was first recognised in late 2019 [2]. At this time (September 2021), it is still spreading globally, and its causative virus, SARS-CoV-2, is mutating into more highly transmissible and conceivably more pathogenic forms. Scientists, at the forefront of COVID-19 pandemic control, have struggled with new concerns not only in the conduct of their research, but also in their roles at the interface of research and research communication.

In this commentary, we address a few of the issues that are germane to preserving the integrity of research and communicating research findings in the context of the COVID-19 pandemic. These issues will likely be familiar to forensic scientists in their daily work, particularly those who provide expert testimony in legal proceedings and communicate with the media. Although our comments emphasise experiences in the US and other developed countries, we stress that the fruits of scientific endeavour must be made available for all. This takes on special significance during a global pandemic, a crisis that touches everyone, and can disproportionately affect those who live in poverty, with limited access to the vaccines, drugs, and treatments that research can provide.

## Integrity in research and public health findings during a pandemic

A deadly pandemic like COVID-19 compels scientists to urgently develop drugs, vaccines, diagnostics, and epidemiologic and clinical knowledge. The sprint to bring efficacious SARS-CoV-2 vaccines to (theoretical) global availability in about 11 months was nearly miraculous, outpacing the previous vaccine development record of about four years. Similarly, polymerase chain reaction (PCR)-based diagnostics were quickly developed and optimized, mass testing was set up, and clinical knowledge was accrued in record time. This included rapid publication of many new discoveries in pre-prints and in prestigious medical and scientific journals. This phenomenon placed knowledge in the public domain almost as soon as the research was completed—in some cases, literally days later. Progress has been slower with treatments and understanding the natural history and pathogenesis of COVID-19, for example, but is still being made.

The accelerated pace of research during a pandemic gives rise to numerous issues. We touch upon several of them here: (1) the pandemic as a moving target, (2) the reliability of findings and certainty about making conclusions, (3) the importance of collaboration, and (4) being conscious of equity and social justice.

## The pandemic as a moving target

An obvious challenge presented by a new disease like COVID-19 is that, especially in early stages, new data replace existing data almost daily. This presents problems in research. Arduous and time-consuming experimentation may be overtaken by someone else’s findings much more quickly than in other kinds of research. New findings make entire research projects suddenly obsolete. New reagents become available and alter research pathways. These pressures make competition for grants, resources, collaborations, and publications even more fierce, and, in turn, the incremental methods of good science may find themselves on shaky ground. In such a climate, the need for speed must not lead to sloppy science or risky corner-cutting, both of which undermine the integrity and trustworthiness of research. A question emerges: how do we achieve the right balance, especially when the pandemic trajectory is constantly changing?

## The reliability of findings and certainty about making conclusions

Experimental scientists know that things often go wrong, and erroneous conclusions can result. How do we deal with this reality when lives are at risk and when everyone, from politicians to the public, demands immediate answers? This question, much like those that frequently emerge in forensic science, exemplifies the age-old dilemma of weighing odds and consequences when stakes are high and different options abound.

The first two SARS-CoV-2 vaccines to be used (i.e. the two mRNA vaccines produced by Moderna and Pfizer, and then used in the US under emergency use authorization) were based on a platform largely untested for safety and efficacy, in hastily conducted clinical trials, and with little time for follow-up of research participants. The primary reason behind such an approach was that, because so many people were dying each day, standard cautions had to be temporarily set aside.

At the height of the pandemic explosion, the wisdom of the decision to speed delivery of mRNA vaccines into mass public use was not seriously questioned, nor do we question it here. Still, it is likely that the calculus will be viewed differently in hindsight, from a perspective of outcomes yet to be experienced. It must be remembered that the field of public health already possessed reliable knowledge to control the COVID-19 pandemic without a vaccine, through measures such as stringent lockdowns, mask wearing, social distancing, and isolation of the infected and the exposed. By using these measures, some countries have been more successful than others at containing the pandemic. Still, as the pandemic wore on in the US, most decision-makers and members of the public, frustrated with restrictions, preferred incompletely tested vaccines as they became available. As a result, rapidly-developed SARS-CoV-2 vaccines, however efficacious, resulted from a panic response designed to pre-empt the difficult pathway of public health control.

The mRNA vaccines have so far proven reasonably safe from serious complications and have been credited with substantial pandemic control in many countries [3]. The Pfizer-BioNTech COVID-19 vaccine has now been fully approved by the US Food and Drug Administration [4]. However, as of September 2021, new SARS-CoV-2 variants such as the delta variant may in some cases be escaping from vaccine-induced immunity, while some fully vaccinated people are being infected and transmitting to others, at least in some situations [5], apparently up-regulating rather than preventing viral spread. This is an outcome that may or may not have occurred with a more traditional vaccine capable of inducing broader immunity.

As far as basing conclusions on findings that are incomplete, or less than reliable, attempts to identify useful therapeutics during this pandemic have fared less well. This is especially true with regard to repurposing drugs licensed for use in other diseases, such as hydroxychloroquine, used in malaria, or the intestinal parasite medicine, ivermectin. One issue for such existing drugs is that they can be legally used for “off label” purposes by medical providers, enabling politicians and celebrities to freely tout their benefits despite the skepticism of experts who understand the scientific evidence. Unfortunately, in 2020 and 2021, the opinions of non-experts seem to have outweighed the opinions of experts in many people’s minds. Consequently, self-styled “experts” can cause harm by endorsing dangerous non-therapeutic chemicals such as bleach and household disinfectants. Sadly, even a few established scientists have let their enthusiasm overtake scientific evidence, spending their credibility on useless or even harmful practices. Like all physicians, physician-scientists should follow the admonition: *primum non nocere* (above all, do no harm). To what extent, if any, should pandemic emergencies push such tenets aside? As scientists, we must hold ourselves accountable in *all* situations and carefully weigh the implications of our actions and words.

Another important aspect of considering the completeness and reliability of data is the necessity of coming to conclusions about consequential research results. Most scientists are careful to avoid becoming too attached to their own pet theories. But the pressure of a pandemic—with lives and economies at risk, pandemic progression moving at breakneck speed, considerable knowledge gaps, and circulation of numerous viable theories—can potentially lead to dogmatic thinking. Fortunately, this has not so far been a major problem with COVID-19 research. Despite missteps in interpreting data on vaccine and drug efficacy, herd immunity, viral origins, and other contentious issues, the lengthy duration of the pandemic has given us time to correct our understanding as we go along. Maintaining integrity as scientists requires that we constantly scrutinize our findings and openly admit and correct our mistakes as knowledge evolves. Can we continue to hold ourselves accountable in this regard? The urgency of a pandemic heightens our need to ensure that our conclusions are based upon reliable evidence and requires us to remain vigilant as we recommend actions that affect public health.

## The importance of collaboration

Researchers usually engage in noble competition, but collegiality can be threatened by the pressure of a pandemic. The COVID-19 pandemic has generally shown how well scientists can work together, not only collaboratively but also in a complementary fashion, i.e. agreeing to tackle different sides of one problem, as has been the case in vaccine development, and in epidemiologic and clinical work. For-profit vaccine manufacturers have even lent facilities and expertise to produce the vaccines of other companies. Some of the most cutting-edge research with the most important implications has appeared on pre-print servers, long before peer review and publication. Each research team is therefore sharing its results in “real time” with all would-be competitors. This willingness to openly share data suggests that the predicament of a global health crisis can in fact bring out the best in scientists, offering a platform for the honorable pursuit of a common goal. Is this a core ethic, or a temporary situational one? Can we build a durable system of collaboration over competition? If so, how so?

We can no doubt learn from the cohesion of collaborative efforts during the pandemic, particularly around rapidly sharing resources and scientific knowledge. Taking a closer look at some of the barriers to such sharing that typically impede collaborations under “normal” circumstances, such as intellectual property rights, national restrictions, limited resources, and fierce competition could be a first step toward translating lessons from this crisis to future work.

## Being conscious of equity and social justice

Social justice is important not only in the context of providing vaccines, drugs, diagnostics, and medical advances to everyone regardless of nationality, wealth, occupation, and other factors, but also in the context of our research endeavors. This means recruiting a broad spectrum of the public in clinical trials of vaccines and drugs. It also means increasing opportunities for underrepresented researchers to conduct important research.

In the case of vaccine development, the high cost of bringing vaccines to licensure, or even to emergency use authorization, has led vaccine developers, understandably, to first focus on demonstrating efficacy and safety in healthy young adults. This has perhaps inadvertently led to inadequate attention to subgroup membership, and only thereafter proceeding to study the same vaccines, perhaps in different dosages, in children, the elderly, pregnant women, and other populations considered more vulnerable. This issue is still being addressed a year and a half after the pandemic first appeared.

It has become increasingly difficult, if not impossible, to reach everyone in society on an equitable basis. Disparities in access to vaccines, drugs, diagnostics, masks, ventilators, and hospital beds are glaringly apparent. This is always the case in a world divided into “haves” and “have nots”, which demands that we recognised that an unequal world will forever remain unable to fully control a pandemic. While this is not a purely scientific problem, science must function optimally within its societal context. We have no formal international mechanism for quickly bringing research products like vaccines and drugs to the global community. Wealthy nations and others who make these products typically prioritize their own populations, and only then consider whether and how to help others.

What should scientists do? Does the ability to discover and create miraculous solutions entail an obligation to make sure they are shared fairly? If so, how? In addition to publishing in open access journals and sharing broadly with collaborators, are there additional steps scientists can take to fulfil their responsibility to foster a just and equitable society? Education and training for researchers should address these questions, reinforcing an altruistic perspective on the fair distribution of valuable healthcare resources and infusing the research culture with thoughtful consideration of our ethical obligations as members of a global community.

## Communicating research and public health findings with integrity

In a pandemic, scientists often find themselves standing at the interface between science and the public, talking to politicians and policymakers, to the media, and directly to the public. It can be unsettling to assume this role. The modern communications universe is complex and fast moving. At times, there seems to be more interest in shocking and titillating than in providing useful information. Such an environment is alien to the world of evidence-based research.

The decline in print media (newspapers, journals, and informative magazines) over the past 20 years has contributed to weakening public awareness of science. To a great extent, public education and interest in science has been replaced by the rise of social media, breathless press deadlines, and a voracious 24-h news cycle for which “news” is defined as only things that happened in the last few hours. Television has become populated by celebrity science skeptics and deniers, undermining the best of scientific work. Perhaps worse is the reliance of individuals on the internet as a trusted library for conducting “research”, along with social media platforms perpetuating conspiracy theories and other misinformation. Given their strong influence on a susceptible public, these platforms have often stymied the truth and thwarted acceptance of scientific evidence and viable public health measures.

As a result of these and other trends, in many Western countries, especially those in which the public has limited knowledge of science, science authorities have become marginalized. For example, in 1964 when US Surgeon General Luther Terry concluded that cigarettes caused heart disease and lung cancer, and thus required a health warning on cigarette packaging, the public listened [6]. Even smokers accepted the verdict, which led to a gradual, if still incomplete, decline in smoking prevalence. Today, little more than 50 years later, when the current US Surgeon General made the science-supported recommendation that people should get SARS-CoV-2 vaccines, he was resisted and even mocked by tens of millions of Americans, including some politicians and public leaders.

In such an environment, how can researchers effectively communicate science and scientific imperatives? Should scientists serve as activists for truth and scientific knowledge? The suggestions we offer for communicating scientific information reflect principles described in the Singapore Statement on Research Integrity [7] and complement other guidelines relevant to diverse disciplines [8].

Some ideas about communicating research with integrity during a pandemic or other major threat or crisis with integrity during a pandemic or other major threat or crisis include the following:

- Be candid and tell the whole truth
- Stay within your realm of knowledge
- Avoid going beyond the scientific findings and avoid speculation
- Avoid pontificating or being too expansive
- Say where to get further reliable information
- If you don’t know, say so
- If worried, say so, and say what is being done to respond to threats
- Say there will be updates, so stay tuned
- Present both sides fairly
- Avoid false equivalences
- Express respectful messaging for all societal members and subgroups
- Avoid “happy talk”; express the gravity of the situation as it really is
- Avoid provocation and defensiveness
- Don’t allow misquotations—follow up and correct them

In essence, by communicating in a candid and forthright manner, relating all that is known in simple terms, admitting what is *not* known, and why, and what is being done to learn it, the responsible scientist remains humble and allows the science to speak for itself. Noting that recommendations may change as circumstances change, and assuring that continuing updates will be provided, scientists can help foster a sense of trust while acknowledging the mutable nature of scientific fields of discovery. Exhibiting tact, with genuine respect and concern for all societal subgroups, demonstrates attention to social responsibility as a scientist.

Within this context, we briefly consider two examples from the COVID-19 pandemic that illustrate specific issues in integrity of research and research communication.

## Risk messaging: science vs. politics

In February 2020, as the COVID-19 pandemic began to explode in the US, a senior CDC epidemiologist, speaking to public health leaders around the country, stated what by then had become obvious to scientists—that further pandemic explosion was inevitable, and that the country needed to prepare [9]. However, this was not the message political leaders wanted to hear or to endorse. The CDC leader was immediately removed from her position as spokesperson [10]. Public health messaging was soon taken away from science experts and moved into the political realm with predictable consequences that were temporally associated with rising COVID-19 deaths exceeding mortality rates in any other nation [10, 11]. Shocked scientists retreated and seemed unprepared to deal with the situation. Not only had they been silenced, but worse, they had to watch scientific evidence relevant to pandemic decision-making being replaced by harmful unscientific beliefs and political agendas [12]. The damage has yet to be fully repaired. The importance of the role of scientific expertise in decision-making has been broadly accepted for centuries. How could we have lost sight of its importance? What can we do to restore the role of scientific expertise in formulation of public policy?

## The origins of SARS- CoV-2

As a result of the specific messaging circumstances just noted, some US politicians shifted the blame for the pandemic explosion away from prevention policy choices to excoriating the country of alleged pandemic origin—the Peoples’ Republic of China. Predictably, this did nothing to control the pandemic, but instead led to verbal and physical attacks on thousands of Americans, not only Americans of Chinese ancestry, but Asian Americans of other ancestries as well. Such “China-blaming” soon led to political attacks on scientists of any ancestry who were studying the origins of the COVID-19 pandemic. From early in 2020, the evidence clearly indicated that SARS-CoV-2 had arisen from the enormous coronavirus bat reservoir, as had been the case with the two previous SARS epidemics, the MERS epidemic, and other coronavirus emergences [13]. RNA sequencing of, and phylogenetic analyses of SARS-CoV-2 and numerous other bat sarbecoviruses found in natural settings in China and Southeast Asia, made it clear that SARS-CoV-2 was composed of RNA segments similar to many other bat viruses from natural settings, without characteristics of viral manipulation [14]. In short, as has always been true in pandemic emergences, “Mother Nature” was solely to blame. Pointing fingers at each other only made pandemic control more difficult, at the cost of many human lives.

However scientifically sound, such findings represented inconvenient facts in the context of blaming China. These findings were countered by unsubstantiated charges that the virus had been sequenced by Chinese and American bioterrorists, or that there had been a “lab leak” accident associated with dangerous Chinese or Chinese-American experiments [15]. Soon, the international scientists regarded as experts in attempting to prevent SARS-like virus emergences saw their work defunded [13]. Some were attacked with death threats [16]. As of September 2021, accumulating research evidence, stronger scientific consensus, and a greater willingness of scientists to advocate for the critical importance of scientific evidence has begun to change the equation. Nevertheless, much damage has been done, and work vital to understanding and preventing future SARS-like emergences has been set back indefinitely.

This tragedy represents yet another example of the marginalization of science. Is it possible to restore the respect for science that has led to continual global progress in multiple areas for hundreds of years? What can be done, and by whom? Much work remains. Scientists must now, more than ever, work together across disciplines and national boundaries, to do whatever it takes to control the pandemic that still rages among us, and which will continue into the indefinite future.

In summary, the COVID-19 pandemic has presented many research challenges, asking that we urgently respond with our best efforts while operating in a climate of hostility and alienation, with direct attacks on science and scientists. We are asked to communicate what we are doing, while our evidence is often dismissed or rejected. The public’s limited understanding of science makes communicating our findings even more difficult. Decisions about pandemic response remain important, yet the evidence-based approach of science is often disparaged or countered with baseless yet widely popular claims. The credibility of science itself is being challenged. How can we uphold and perpetuate our standards of integrity, both individually and collectively?

Our responses must be active, respectful, and informed by ethical deliberation and rigorous debate. Careful examination of these kinds of questions, besides being incorporated in research mentoring and formal training, should also occur in broader settings that engage diverse scientists and members of the global community that our scientific results are designed to serve.

## Authors’ contributions

Both authors contributed equally to the planning, writing and editing of the manuscript.


David Morens
*National Institute of Allergy and Infectious Diseases, National Institutes of Health, Bethesda, Maryland, USA*

dmorens@niaid.nih.gov

Zoë Hammatt
*University of Hawaii and Z Consulting, Honolulu, Hawaii, USA*


